# Cytokine profile and viral diversity in the early seronegative stage of community-acquired hepatitis C virus (HCV) infection

**DOI:** 10.1038/s41598-023-47335-x

**Published:** 2023-11-16

**Authors:** Marek Radkowski, Piotr Grabarczyk, Tomasz Kryczka, Kamila Caraballo Cortès, Dorota Kubicka-Russel, Maciej Janiak, Sylwia Osuch, Karol Perlejewski, Tomasz Laskus

**Affiliations:** 1 Department of Immunopathology of Infectious and Parasitic Diseases, Medical University of Warsaw, Warsaw, Poland; 2 Department of Virology, Institute of Hematology and Transfusion Medicine, Warsaw, Poland; 3 Department of Development of Nursing and Social and Medical Sciences, Medical University of Warsaw, Warsaw, Poland; 4 Department of Adult Infectious Diseases, Medical University of Warsaw, 37 Wolska St., 01-201 Warsaw, Poland

**Keywords:** Immunology, Microbiology, Gastroenterology, Pathogenesis

## Abstract

Most Hepatitis C virus (HCV)-infected subjects develop chronic infection, whereas a minority clear the virus in the early phase of infection. We analyzed factors associated with outcome (chronicity vs clearance) during the preclinical seronegative phase of community-acquired HCV infection. Among 17.5 million blood donations in the years 2000–2016, 124 blood donors were found to be HCV RNA-positive/anti-HCV-negative. All were contacted after 0.5–12.7 years and 40 responded and provided blood sample. Hypervariable region 1 was analyzed by ultradeep pyrosequencing and cytokines in serum were quantified by Luminex (R&D Systems) multiplex immunoassay. Twenty-one (52.5%) donors were found to be HCV-RNA-positive, while 19 (47.5%) were HCV RNA negative (none received antiviral treatment). All but one seroconverted to anti-HCV. Donors with resolving hepatitis did not differ significantly from donors with chronic infection with respect to age, genotypes, IL28B polymorphisms, number of viral variants, nucleotide diversity per site or the overall number of nucleotide substitutions. However, the former group had significantly higher levels of IL-1beta, IL-1RA, IL-6, IFN-gamma and FGF-2 in serum. In our study of community-acquired acute hepatitis C approximately half of all subjects eliminated the virus spontaneously, and this clearance was associated with marked cytokine response in the early seronegative stage of infection.

## Introduction

Hepatitis C virus (HCV) infection is a common etiologic factor of chronic hepatitis, liver cirrhosis, and hepatocellular carcinoma (HCC)^1^. The World Health Organization’s Global Hepatitis Report estimates that about 71 million individuals are currently infected with HCV worldwide^2^, but other estimates put the number of infected as high as 118.9 million^3^. However, reliable data on HCV prevalence are difficult to obtain as surveillance studies on representative population are rare and usually do not include individuals with high risk factors such as homelessness or incarceration. In the United States, the estimated overall prevalence of anti-HCV in the adult population is 1.7% (indicating past or current infection), and approximately 2.4 million Americans carry the virus^4^. These numbers are based on the National Health and Nutrition Examination Survey (NHANES) and data from high-risk underrepresented groups. Similar HCV prevalence has been reported in Western Europe and Poland^2^. The majority of infected subjects (55–80%) develop chronic infection, whereas only a minority clear the virus almost exclusively in the early phase of infection^2^.

It is the effectiveness of host immune response, particularly of specific CD4^+^ and CD8^+^ T-cells and production of effector cytokines that determine the outcome of HCV infection^5–7^. However, as the HCV infection continues, there is gradual impairment of the host immune function, referred to as immune exhaustion, and spontaneous clearance of the virus becomes very unlikely^8^. Furthermore, it is likely that rapid escape mutations may outpace the immune system and thus facilitate viral persistence. This scenario is supported by observations that resolving acute hepatitis is typically characterized by relative evolutionary stasis, while chronic infection is associated with genetic evolution in the first months of infection^9,10^. The critical role of effective immune response is also validated by observations that transplant recipients receiving immunosuppressive drugs almost uniformly develop chronic infection when exposed to HCV and spontaneous clearance of the virus in this setting is an extremely rare event^11^.

Studies on the natural course of infection from acute preclinical phase to resolution or chronicity are extremely limited, mainly owing to difficulties in obtaining samples from patients during the early phase of primary infection, and thus are largely confined to chimpanzee studies and iatrogenic exposures such as transfusions and needlestick accidents among health care workers^6,12–16^. However, the latter studies may not be representative for the general population, as the most common form of HCV infection is not iatrogenic but community-acquired. Furthermore, since only 58% of subjects with community-acquired infection were found to be HCV RNA positive^4^, they may have a better prognosis overall than iatrogenic infections, where chronicity rate is close to 80%^12–14^. We had a unique opportunity to study a large group of subjects identified in the early preclinical seronegative phase of HCV infection and to analyze factors associated with long-term outcome (chronicity vs infection clearance).

## Patients

In the years 2000–2016 there were over 17.5 million donations collected in 23 Blood Transfusion Centers in Poland. One hundred twenty-four donors were found to be HCV RNA positive and anti-HCV negative at the time of donation. All these donors were contacted by respective Blood Transfusion Centers 0.5–12.7 years after their HCV-positive donation, with 40 responding and providing a follow-up blood sample. These 40 patients constituted the study group.

Eight (20%) were first time donors while the remaining 32 (80%) were repeat donors and the time from their last donation was no longer than 6 months. All were negative for markers of HBV and HIV infection. None of these 40 donors received antiviral treatment prior to the current study. In a standard questionnaire required before each donation, all donors denied any risk behaviors; however, once identified as infected and called within 1–2 weeks for a more detailed interview 12 (30%) admitted to unprotected sex with a new partner(s) within the last 6 months and four of them also admitted to injecting drugs in this period. Two more donors had nonsexual contacts with an infected family member, while in the remaining 26 (65%) donors no risk factors could be identified.

Out of the remaining 84 HCV RNA positive donors, only 44 came for the interview. High-risk sexual behaviour was the most common risk factor as it was admitted by nine donors (20%). Six of them had unprotected sex with a new partner(s) and another three had unprotected sex with somebody known to be HCV-positive. Five more admitted to injecting drugs, four had persistent nonsexual contacts with an infected family member, and two were incarcerated. No risk factors were admitted by the remaining 24 (55%) donors.

## Results

Forty patients responded to the call and provided follow-up samples. In 21 (52.5%) subjects, HCV-RNA screening performed with follow-up serum samples revealed virus presence, while the remaining 19 (47.5%) were negative (none of them received antiviral treatment) fulfilling the criteria of spontaneous virus elimination. The mean time between the baseline (index sample) and follow-up sample was 4.3 years (median: 2.4, range: 0.5–12.7 years). This time was over 10 years in 8 donors, 4–10 years in seven donors, 1–4 years in 17, and 6–12 months in remaining eight donors. The mean length of time between donation and follow-up for the 84 donors who did not respond was 4.5 years (median 2.7 years). All but one donor seroconverted to anti-HCV in the follow-up period; this one donor was HCV RNA negative in the follow up sample drawn 8.9 years after the index donation. Clinical, virological and laboratory data in both groups of donors are presented in Table 1. The 84 patients who did not respond did not differ from the responders with respect to age (26.5 ± 8.1 years vs 28.0 ± 11.1 years; NS) but were more likely to be male although this difference did not reach statistical significance (91% vs 78%; p = 0.09).

**Table 1:** Some clinical, virological, biochemical, and immunological data in 19 blood donors who spontaneously cleared HCV infection compared with 21 donors who remained infected.

		Resolved infection (N = 19)	Chronic infection (N = 21)	p value
M/F		12/7	19/2	0.06
Age (years), mean ± SD		26 ± 7.4	27 ± 8.7	0.64
First time donation		5	3	
Any risk factor present		7	7	
Viral load (× 10^6^ IU/mL), mean ± SD		5.5 ± 17.7	1.7 ± 2.8	0.85
Genotype	1b/1a	8	9	
	3	9	8	
	4c/4d	0	4	
	Other/undetermined	2	0	
HVR1 variability^a^ (mean ± SD)	No. of variants	4.4 ± 1.8	4.75 ± 3.2	0.81
	Nucleotide diversity per site π	0.057 ± 0.067	0.026 ± 0.039	0.19
	No. of substitutions	22 ± 25	12 ± 17	0.29
IL-28B	C/C	2	2	1
	T/T	4	6	0.7
	C/T	9	8	0.72
Baseline ALT^b^ (mean ± SD)		55.2 ± 57.4	99.53 ± 144.60	0.1090

^a^With respect to consensus sequence (the most represented sequence within the sample).

^b^ALT range 6–50 (U/L) reference.

Twenty-one donors with resolving hepatitis did not differ significantly from 19 donors with chronic infection with respect to age, but the proportion of females in the former group was higher and close to being statistically significant (37% vs 10%; p = 0.06). Similarly, the distribution of infecting genotypes was not different in both groups. Consistent with the distribution of HCV genotypes in Poland, the majority of patients were infected with type 1b or 1a strains followed by genotype 3; 4 donors were infected with genotype 4 and in 2 the genotype could not be determined. In all HCV-RNA positive follow-up samples the viral genotype was the same as in the respective initial serum samples.

When HCV HVR1 variability was analyzed by NGS in the baseline index sample, the number of identified viral variants, nucleotide diversity per site, and the overall number of nucleotide substitutions were similar in both groups. Furthermore, donors with resolving and chronic infection did not differ with respect to IL28B polymorphisms (Table 1). Interestingly, patients who eventually progressed to chronicity had markedly higher ALT activity already in the early seronegative phase of infection, but this difference did not reach statistical significance (Table 1).

However, analysis of cytokines/chemokines in serum revealed that the former group had significantly higher levels of IL-1beta, IL-1RA, IL-6, IFN-gamma and FGF-2 (Table 2).

**Table 2:** Serum cytokines in 19 blood donors who spontaneously cleared HCV infection compared with 21 donors who remained infected.

Serum cytokine pg/mL (mean ± SD)	Resolved infection (N = 19)	Chronic infection (N = 21)	p value
Eotaxin	60.99 ± 34.43	54.11 ± 21.39	0.8911
FGF-2	13.90 ± 21.39	1.29 ± 0.55	0.0115
IL-1beta	25.32 ± 17.58	13.68 ± 4.38	0.0249
IL-1RA	1228 ± 618.10	528.60 ± 233.40	0.0008
IL-5	50.91 ± 40.75	30.18 ± 2.00	0.7596
IL-7	13.24 ± 2.45	12.54 ± 7.50	0.9300
IL-6	45.43 ± 60.51	1.31 ± 0.32	0.0036
IL-8	0.54 ± 0.34	0.36 ± 0.36	0.0832
IL-10	15.56 ± 45.89	0.54 ± 1.10	0.5232
IL-12p70	18.36 ± 18.12	12.34 ± 4.44	0.7596
IP-10	55.14 ± 35.10	82.33 ± 87.48	0.2846
MIP-1 alpha	4.78 ± 7.27	4.86 ± 7.18	0.9366
PDGF-BB	365.40 ± 469.10	158.90 ± 171.90	0.8221
TNF-alpha	23.56 ± 19.92	11.22 ± 5.82	0.1084
G-CSF	116.4 ± 83.88	67.91 ± 21.31	0.2617
GM-CSF	16.43 ± 27.72	0.52 ± 0.29	0.0860
IFN-gamma	16.05 ± 32.94	1.91 ± 0.15	0.0453

## Discussion

While chronic hepatitis C is common and has been the subject of numerous studies, very few patients are identified during the early stage of infection, and the vast majority of them only because they are symptomatic. However, absent a surveillance program such as following an iatrogenic or occupational exposure, asymptomatic infections, which constitute the vast majority of de novo HCV infections, elude detection or are detected accidentally. Thus, the most common form of HCV infection, which is community-acquired and asymptomatic, is largely missing from published studies, except for an occasional evaluation of injection drug users^17^. In the current analysis we took advantage of a large-scale blood donations screening to identify donors in the early stage of HCV infection: they were HCV RNA positive but anti-HCV negative and the previous donation within the preceding 6 months was negative for both of these infection makers. Screening of 17 million donations allowed for the identification of 124 such donors and 40 of them could be traced down and provided a serum sample for the determination of HCV status. The current study is the largest analysis of community-acquired sporadic HCV infections identified in the pre-seroconversion phase so far, and one of the largest with regard to the number of acute hepatitis cases analyzed^18^.

In our study 50% of followed up donors were found to be HCV RNA negative and thus were considered to have cleared the infection spontaneously. In a systematic review of 31 acute hepatitis studies, Micallef et al*.*^18^ reported that only 26% of all patients cleared the infection and patients with clinical hepatitis had a significantly higher proportion of viral clearance (31%) than those in both post-transfusion (18%) and sero-incident studies (18%). Similar results were reported by Gerlach et al*.*^19^ in their large study of 60 cases of acute hepatitis: while spontaneous clearance was observed in 52% of acute symptomatic cases, all asymptomatic patients developed chronic infection. However, the latter group was small as it encompassed only 9 patients. As an explanation of the above phenomenon, it was proposed that symptomatic disease is a surrogate marker of the strength of the immune response^20^. The reason for the discrepancy in the clearance rate of asymptomatic infections between above reports and the current study is unclear, but our data are in line with more recent population-wide studies in which only slightly over half of anti-HCV positive patients were found to be HCV RNA positive^4^. Perhaps the somewhat higher clearance rate of community-acquired infections could be related to lower inoculation viral loads resulting in a more pronounced ‘bottleneck effect’, when compared to transfusion-related and iatrogenic exposures.

While all donors initially denied any risky behavior, once identified as infected and questioned in a face to face interview, 12 (30%) admitted to unprotected sex with new partner(s) within the last 6 months (four of them also injected drugs within this time). These donors were likely so called 'test seekers', as blood donations are often the cheapest and simplest way to get tested for HIV and other transmissible diseases in a socially acceptable and non-stigmatizing setting^21^. Thus, our study suggests a significant role of sexual transmission in the community acquired HCV infection and likely horizontal transmission of HCV from spouse to spouse has been reported in a number of studies^22–26^. However, the latter evidence is mostly circumstantial and an increased risk of transmission from HCV-positive patients to household members also includes siblings, parents, and offspring, which suggests that long and close contact represent a risk in itself^27–30^. Most cases of new HCV infection identified currently in Western Europe and USA have been associated with a history of injecting drug use and iatrogenic exposure in health-care facilities, but in a large proportion of cases no clear risk factor can be identified^19,31,32^.

There is evidence from chimpanzee and iatrogenic exposure studies that early events determine the eventual outcome of infection—clearance vs chronicity^5,6^. Our findings suggest that this is also true for community-acquired infections as the subjects who cleared infection had significantly higher levels of IL-1, IL-6, FGF2, and IFN-gamma.

Viral infections can be cleared in two ways—by the destruction of infected cells via the natural killer (NK) cells of the inane immune system and cytotoxic T cells (CTL) of the adaptive immune system and in the noncytolytic process mediated by antiviral cytokines^33^. As our subjects who cleared infection had somewhat lower ALT activity and higher cytokines’ levels, including IFN-gamma, it suggests that the latter was the dominant factor. It is well established that virus-specific CD8+ T cells are required for the control of HCV infection, and their effector function includes both cytolytic and non-cytolytic mechanisms. The latter, which is IFN-gamma mediated, seems to be dominant as in an in vitro model it was responsible for > 95% viral inhibition at low effector-to-target ratios where no cytotoxicity was observed^34^. IFN-gamma production seems also to be an important component of early NK cell response to HCV exposure^35^ and its production is increased in acute^36^, but decreased in chronic hepatitis C, probably facilitating the inability to eradicate the virus^37^.

While not directly antiviral, IL-1 (IL-1 alpha, IL-1 beta, IL-1Ra) and IL-6 are considered important cytokines produced in response to infection by macrophages, T and B cells, dendritic cells, but also by multiple other cell types^38^. IL-6 is a very pleiotropic activating JAK/STAT3 pathway, promoting transcription of genes involved in cellular signaling and gene regulation and has both pro- and anti-inflammatory effects^39^. Patients clearing infection also had higher levels of fibroblast growth factor 2 (FGF2). Growing evidence suggests that the fibroblast growth factor/FGF receptor (FGF/FGFR) signaling plays a significant role in regulating cellular lineage commitment, differentiation, proliferation, and apoptosis of various types of cells^40,41^. FGF2 itself is pro-inflammatory and increased serum levels were previously described in patients with chronic HCV infection, particularly in those with high viral loads^40,41^.

Several studies reported on the presence of cellular reactivity in the absence of anti-HCV, suggesting the existence of some form of transient and likely localized infections^42,43^. However, among our subjects, all but one out of 40 seroconverted, which suggests that once there is a detectable level of replication, seroconversion is almost universal. Our data are in line with a large prospective analysis of HCV infection in IDU, in which HCV RNA could not be detected in a subset of cohort who never seroconverted^17^. Similarly, HCV-specific antibodies were not induced in the absence of viremia in a prospective analysis of 12 health care workers accidentally exposed to low amounts of HCV^35^. Despite being persistently HCV RNA negative, the subjects in the latter study showed evidence of specific NKT/NK cells, chemokines and T-cells response.

We did not find any correlation between infection outcome and HCV E2/HVR1 quasispecies divergence or complexity; however, dynamics were not studied as only a single time-point serum sample was available. In several studies, changes in E2/HVR1 quasispecies during the acute phase of infection, likely caused by mounting immune pressure, were predictive of ensuing chronic infection, whereas stability was associated with resolution^9,10^. Patients carrying an IL28B homozygote for the major alleles of rs12979860 (CC genotype) were reported to have enhanced spontaneous HCV clearance rate^44^, but this genotype was rare among our patients as it was present in only four patients, two of which cleared infection. Similarly, while some studies reported that higher initial viremia is associated with spontaneous clearance^45^, we did not observe any differences in the serum viral loads between patients who cleared the infection and those who remained HCV RNA positive.

In summary, in our study of community-acquired acute hepatitis C, we found that approximately half of all subjects eliminate the virus on their own, and this clearance is associated with marked cytokine response in the early seronegative stage of infection.

## Methods

The study was conducted in accordance with both the Declarations of Helsinki and Istanbul, and informed consent was obtained from all subjects. The study was approved by the Bioethical Committee at the Institute of Hematology and Transfusion Medicine, Warsaw, Poland (no. 36/2016).

### Blood screening for HCV

The pre-donation HCV RNA testing before 2005 was performed in minipools of 48 using Cobas Amplicor HCV v 2.0 (Roche Molecular Systems, Inc., Branchburg, USA), in the years 2005–2006 in minipools of 24 using Cobas Ampliscreen v 2.0 (Roche) and since 2007 in minipools of 6 samples using first Cobas Taqscreen MPX (Roche) and after 2012 using Cobas Taqscreen MPX ver 2 assay (Roche). Individual donation testing was performed employing transcription mediated assays (TMA): before 2005 by Procleix HCV/HIV-1 (Gen-Probe Incorporated, San Diego, USA), in the years 2005–2009 by Procleix Ultrio (Gen-Probe Incorporated), in the years 2010–2012 by Procleix Ultrio Plus (Gen-Probe Incorporated) and since 2013 using Procleix Utrio Ellite (Gen-Probe Incorporated).

Anti-HCV testing was performed by one of the following immunoenzymatic assays: HCV ELISA V3.0 (Ortho-Clinical Diagnostics, Inc a Johnson & Johnson Company, Raritan, USA), Architect Anti-HCV (ABBOTT, Wiesbaden, Germany) and Vitros aHCV (Ortho-Clinical Diagnostics, Wycombe Buckinghamshire, United Kingdom).

### HCV RNA amplification

A non-commercial PCR spanning 252 bp-long fragment of viral 5’UTR (sensitivity ≤ 10 genomic/Eq) was used to detect viral RNA^10^ and HCV RNA load was quantified using by Cobas TaqMan HCV Test (Roche Molecular Systems, Branchburg, NJ, USA), which has a limit of detection between 10 and 20 IU/mL and a lower limit of quantification of 25 IU/mL.

### Amplification of hypervariable region 1 (HCV HVR1)

Total RNA was extracted from 250 μL of serum by a modified guanidinium thiocyanate phenol/chloroform method using Trizol (Life Technologies, Carlsbad, CA, USA) and suspended in ten µL of water. In the next step 5.65 µL of RNA was subjected to reverse transcription process at 42 °C for 60 min using AccuScript High Fidelity Reverse Transcriptase (Agilent Technologies, Santa Clara, CA, USA) and Random Hexamers (Invitrogen; Carlsbad, CA, USA). For HCV genotype 1 primers used in the first step were as follows: 5′-GGTGCTCACTGGGGAGTCCT-3′ (nt 1389–1408) and 5′-CATTGCAGTTCAGGGCCGTGCTA-3′ (nt 1632–1610). PCR was carried out using 2.5 U Fast Start High Fidelity Enzyme Blend (Roche Diagnostics, Indianapolis, IN, USA), 1× buffer, 1.8 mM MgCl_2_, 0.2 mM dNTP and 40 pmol of each primer in a total volume of 50 µL. Five µL of cDNA was added to the PCR mixture. The thermal profile for the amplification was as follows: initial denaturation at 95 °C for 7 min; 45 cycles with denaturation at 94 °C for 30 s, annealing at 55 °C for 25 s and elongation at 72 °C for 30 s. The reaction was terminated with a final elongation at 72 °C for 7 min.

Second step PCR was performed with 5 µL of first PCR product and primers containing multiplex identifiers (MID), specific for each sample, recognized by GS Junior sequencing platform. The sequence-specific primers were as follows: 5′-TCCATGGTGGGGAACTGGGC-3′ (nt 1428–1447) and 5′-TGCCAACTGCCATTGGTGTT-3′ (nt 1603–1584). Thermal profile for the amplification was as follows: initial denaturation at 95 °C for 7 min; 30 cycles with denaturation at 94 °C for 30 s, annealing at 60 °C for 1 min and elongation at 72 °C for 30 s. The reaction was terminated with a final elongation at 72 °C for 10 min.

For HCV genotype 3 primers used in the first step were as follows: 5′-ATGGCATGGGATATGAT-3′ (nt 1291–1307) and 5′-AAGGCCGTCCTGTTGA-3′ (nt 1619–1604). Thermal profile for the amplification was as follows: initial denaturation at 95 °C for 7 min; 45 cycles with denaturation at 95 °C for 45 s, annealing at 55 °C for 1 min and elongation at 72 °C for 1 min. The reaction was terminated with a final elongation at 72 °C for 10 min.

Second step primers were as follows: 5′-GGCAACTGGGCCAAGGTCGC-3′ (nt 1437–1456) and 5′-ATGTGCCACGAGCCATTGGT-3′ (nt 1606–1587). Thermal profile for the amplification was as follows: initial denaturation at 95 °C for 7 min; 30 cycles with denaturation at 94 °C for 45 s, annealing at 60 °C for 1 min and elongation at 72 °C for 1 min. The reaction was terminated with a final elongation at 72 °C for 10 min.

### Ultradeep pyrosequencing

Amplicons were purified from agarose gel by QIAquick Gel Extraction Kit (Qiagen, Hilden, Germany) and Agencourt AMPure XP purification system (Beckman Coulter, Beverly, MA) then measured fluorometrically using Quant-iT High-Sensitivity dsDNA Assay Kit (Life Technologies, Carlsbad, CA) on Qubit 3.0 Fluorometer (Life Technologies, Carlsbad, CA). Samples were pooled in equivalent amounts and 3 × 107 amplicons were subjected to emulsion PCR using GS Junior Titanium emPCR Lib-A Kit (454/Roche, Branford, CT, USA). Pyrosequencing was performed following the amplicon processing protocol for 100 cycles using full processing mode for amplicons according to manufacturer’s instructions using GS Junior System (454/Roche; Branford, CT, USA).

### Sequencing data analysis

Sequencing errors (insertions, deletions and mismatches) were corrected and haplotypes inferred with use of diri_sampler program from the Shorah software (https://www1.ethz.ch/bsse/cbg/software/shorah); (1). Haplotypes with posterior probability > 95% represented by at least 10 reads were extracted with LStructure (https://github.com/ozagordi/LocalVariants/blob/master/src/LStructure.py).

Based on pyrosequencing and reconstruction of HCV HVR1 sequence^46,47^, it was possible to credibly detect viral variants constituting as little as 1% of the population and this cut-off value was implemented in further analysis. In the next step, haplotypes were aligned to the HCV reference sequences (GenBank accession number AJ406073 for genotype 1b, EF560242.1 for genotype 1a and EF442261.1 for genotype 3) and trimmed to be equal in length and alignment. Subsequently, genetic diversity parameters (i.e. intrahost number of variants, number of nucleotide substitutions and nucleotide diversity per site π) were estimated by DNA SP version 5^48^ and MEGA 5.0^49^.

The number of nucleotide substitutions and nucleotide diversity per site π, representing the average number of nucleotide differences per site, were calculated with respect to consensus sequence (the most represented sequence within the sample).

### Quantification of cytokines and chemokines

Luminex (R&D Systems Inc., McKinley Place NE, MN, USA) multiplex immunoassay was used for quantitative detection of 24 cytokines, chemokines and growth factors (eotaxin, FGF-2, G-CSF, GM-CSF, IFN-gamma, IL-1beta, IL-1RA, IL-10, IL-12p70, IL-13, IL-17A, IL-4, IL-5, IL-6, IL-7, IL-8, IL-9, IP-10, MIP-1alpha, MIP-1beta, PDGF-BB, TNF-alpha, TNF-beta, VEGF-A) in patients baseline serum samples according to manufacturer instructions.

### IL28B

The IL28B polymorphisms (rs12979860, rs8099917) were analyzed using the TaqMan SNP genotyping assay (Applied Biosystems Inc, Foster City, CA, USA) using the 7500 Fast real-time thermocycler. Genotyping calls were verified with SDS software (Applied Biosystems Inc.).

### Statistical analysis

Statistical analysis was performed using GraphPad Prism ver. 9.0. The values were compared by the nonparametric Mann–Whitney U test whereas frequency variables were compared by Fisher exact test.

## Data Availability

The datasets generated and analyzed during the current study are available from the Sequence Read Archive (PRJNA997777) and clinical data are available from the corresponding author on reasonable request.
